# Circadian systems biology: When time matters

**DOI:** 10.1016/j.csbj.2015.07.001

**Published:** 2015-07-17

**Authors:** Luise Fuhr, Mónica Abreu, Patrick Pett, Angela Relógio

**Affiliations:** a Institute for Theoretical Biology (ITB), Charité-Universitätsmedizin Berlin and Humboldt-Universität zu Berlin, Invalidenstraße 43, 10115 Berlin, Germany; b Molekulares Krebsforschungszentrum (MKFZ), Charité-Universitätsmedizin Berlin, Augustenburger Platz 1, 13353 Berlin, Germany

**Keywords:** Circadian clock, Cancer, Mathematical modelling, Chronobiology

## Abstract

The circadian clock is a powerful endogenous timing system, which allows organisms to fine-tune their physiology and behaviour to the geophysical time. The interplay of a distinct set of core-clock genes and proteins generates oscillations in expression of output target genes which temporally regulate numerous molecular and cellular processes. The study of the circadian timing at the organismal as well as at the cellular level outlines the field of chronobiology, which has been highly interdisciplinary ever since its origins. The development of high-throughput approaches enables the study of the clock at a systems level. In addition to experimental approaches, computational clock models exist which allow the analysis of rhythmic properties of the clock network. Such mathematical models aid mechanistic understanding and can be used to predict outcomes of distinct perturbations in clock components, thereby generating new hypotheses regarding the putative function of particular clock genes. Perturbations in the circadian timing system are linked to numerous molecular dysfunctions and may result in severe pathologies including cancer. A comprehensive knowledge regarding the mechanistic of the circadian system is crucial to develop new procedures to investigate pathologies associated with a deregulated clock.

In this manuscript we review the combination of experimental methodologies, bioinformatics and theoretical models that have been essential to explore this remarkable timing-system. Such an integrative and interdisciplinary approach may provide new strategies with regard to chronotherapeutic treatment and new insights concerning the restoration of the circadian timing in clock-associated diseases.

## 1 Introduction

The ability to adapt to and anticipate the light/dark cycles of the earth offered a survival advantage to many organisms that prevailed throughout evolution. As such, most organisms evolved an endogenous timing system—the circadian clock.

The circadian clock drives numerous physiological and behavioural processes, which consequently follow a rhythm of approximately 24 h and ensure an accurate adaptation to external daily rhythms [1]. In the last decades the field of chronobiology, which studies these biological rhythms, has boomed. As a consequence, theoretical and experimental tools were developed which fostered research and discovery in the field.

**Fig. 1:**
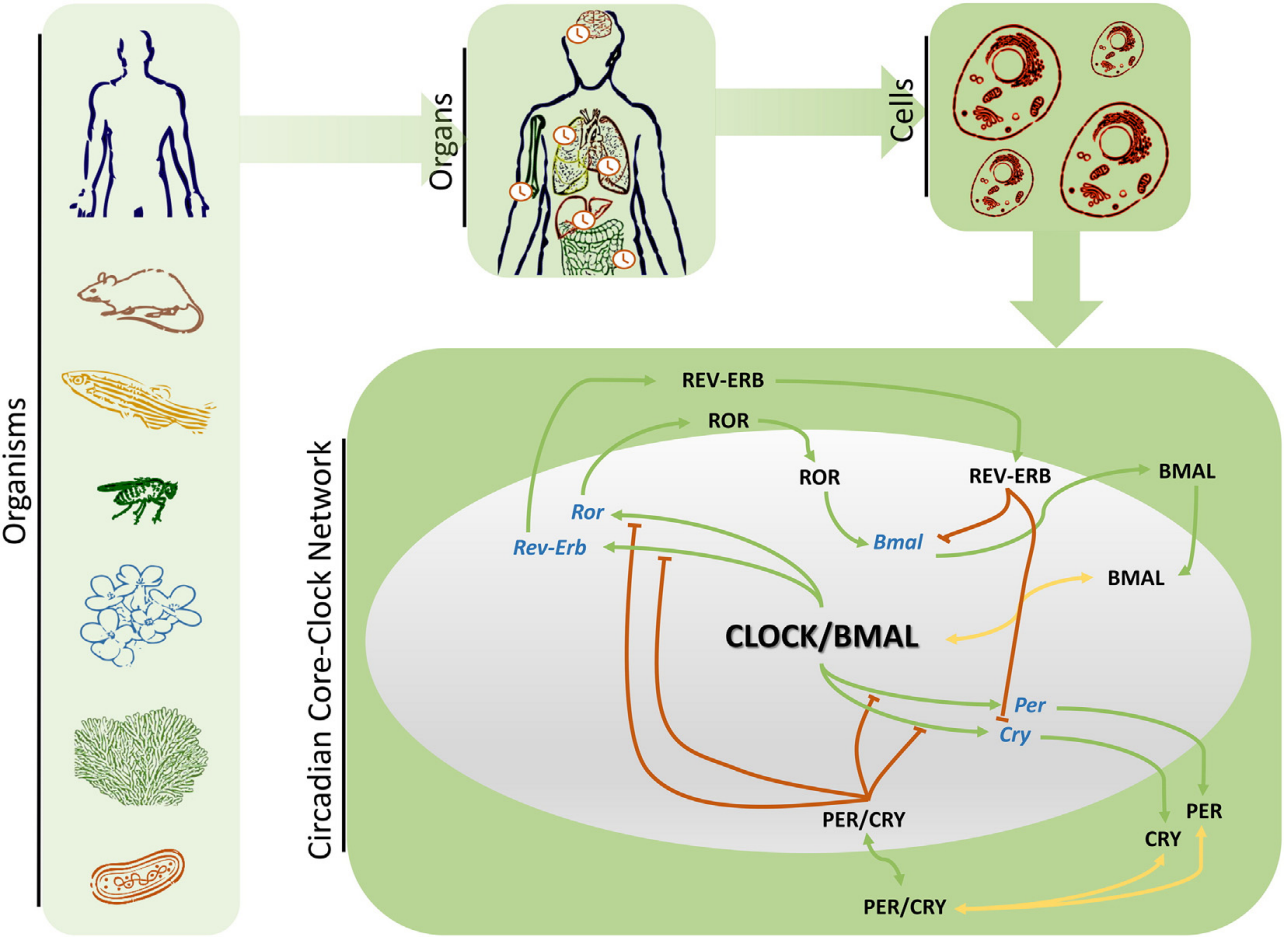
Circadian systems biology. The circadian clock is present in a large variety of organisms from simple unicellular organisms to complex mammalian systems. In mammals, a main pacemaker is located in the SCN, and peripheral clocks exist in each organ which regulate the timing of physiological processes. In fact, every single cell has its own clock and all these individual clocks are synchronized and entrained to signals received from the main pacemaker. At the cellular level the complex—CLOCK/BMAL—regulates a set of positive (green) and negative (orange) interactions which form feedback-loops. These feedback-loops are accountable for the generation of oscillations in gene expression.

Circadian research has been extremely interdisciplinary and has attracted researchers from various scientific backgrounds. It was the astronomer Jean Jacques d'Ortous deMairan, who in 1729 provided evidence for the existence of circadian rhythms. He noticed that the daily leaf movements of the heliotrope plant, *Mimosa pudica*, persisted in constant darkness and suggested the existence of an endogenous time-generating mechanism, in line with the geophysical time [2]. However, the first evidence for a genetic basis of circadian rhythms was provided two centuries later, by Bünning. He reported that in common beans, the period lengths of the offspring ranged between the extremes of period lengths of the parent generation [3]. During the 20th century circadian rhythms were reported and further studied in other organisms as well, from cyanobacteria to humans (Fig. 1) [4].

Even though circadian research started centuries earlier, it was only in 1959 that, for the first time, Halberg used the term circadian (*circa* and *dies* “about a day”) to describe the observed rhythms [3]. Shortly thereafter, in 1960, the Cold Spring Harbour Symposium on Biological Clocks [5] brought together researchers working on circadian rhythms, from experimental to rather mathematical and computational backgrounds. This event established the basis of circadian investigation and paved the way to chronobiology [3].

From thereafter, the search for the “clock” started. In 1971, Konopka and Benzer identified the first clock gene—*Period*—in *Drosophila melanogaster* by an EMS-induced mutant screen [6]. The following milestone in circadian rhythms research was the identification of the suprachiasmatic nucleus (SCN)—a brain region located in the hypothalamus—as the central pacemaker of the circadian clock, in 1990 [7].

Meanwhile, 14 core-clock genes [8] were identified in mammals and shown to form complex transcriptional/translational networks, that are able to drive oscillations in gene expression of output target genes, the clock-controlled genes. It became apparent that the circadian clock has systemic effects at the organismal level which are due to the intricate dynamics of this rather small number of core-clock genes generating a highly precise signal and leading to the propagation of a cascade of events which ultimately influence numerous cellular processes. Within these lines, our group and others have identified new clock-controlled genes and generated elaborate networks of circadian regulation for mammals [9–11].

**Fig. 2:**
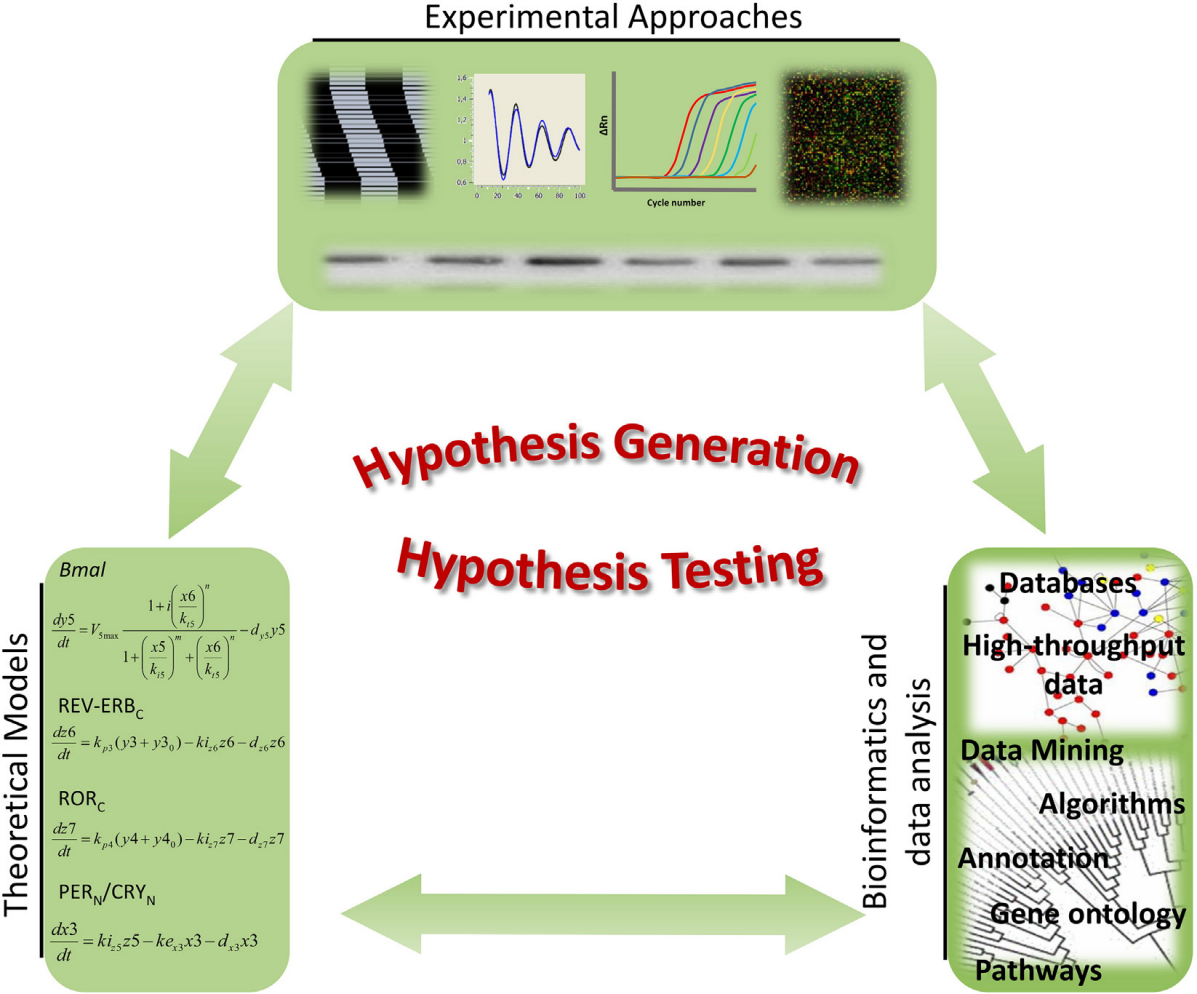
Measuring circadian rhythms. The oscillatory output of a model system can be observed in experimental models and simulated using computational approaches. Experimentally, different sorts of data may be produced to quantify circadian rhythms, depending on the question addressed and model used. These include, for example, actograms, bioluminescence live-cell recordings, RT-qPCR, microarray and western blot data. Also computationally, different methods are available to investigate the circadian system, mostly based on ordinary differential equations (ODEs). By means of bioinformatics approaches the analysis of experimental data and also the integration of experimental and computational data can be carried out. The interdisciplinary nature of circadian research is here once more present.

Systems biology approaches are crucial to investigate the clock on a systems-wide level as shown in Fig. 2. The usage of large-scale component identification using genome-wide technologies, and the development and implementation of *in silico* mathematical models, able to integrate multiple levels of data and generate predictions and testable hypotheses, are the key building blocks in systems biology which are being transferred to circadian systems biology. Nevertheless, targeted smaller scale experimental approaches are essential to validate results gained from systems biology approaches. Since the 1990s, genomics, proteomics and metabolomics data generation dramatically developed. High-throughput methods such as microarrays, next-generation sequencing and mass spectrometry now allow for the global analysis of cells, tissues and organisms and require models and tools to analyse such massive datasets [12,13]. In parallel, mathematical models were developed and are widely used to simulate the complexity of processes involved in the generation of circadian rhythms [14].

In this review, we focus on what started as a rather unusual, not wide accepted field of research and succeeded in demonstrating that time matters and that deregulation of the temporal system has severe consequences in disease and therapy. Nevertheless, it is important to point out that the circadian system influences many other fields apart from medicine which include agriculture, psychology, ecology and environmental biology [15–18].

In the following, we provide an overview of the circadian clock, with a focus on the mammalian clock, the established methods to study it experimentally, as well as theoretically and the consequences, at the organism level, of the failure of this remarkable time-generating system.

## 2 Basic structure of the circadian system: the core-clock

The mammalian circadian clock is hierarchically organized in three main components: input signalling pathways, the main pacemaker (or central oscillator) and output signalling pathways.

Signals received from the environment are communicated via the input pathway to the main pacemaker. These signals, named zeitgebers or timing cues, are used to synchronize the pacemaker oscillations with the solar day–night cycle. Light is consequently the strongest zeitgeber, but also temperature, noise, food, exercise and melatonin can act as zeitgebers [19].

The central oscillator is formed by two clusters of neurons (~ 100,000 neurons in each cluster in humans) and is located in the hypothalamus above the optic chiasm, thus named Suprachiasmatic Nucleus (SCN) [1].

Light signals are received by the retinal cells which transmit the input to the SCN via the retino-hypothalamic tract. In the SCN, calcium influx occurs in response to the interaction of glutamate with NMDA (N-Methyl-D-aspartate) receptors, leading to the activation of IP3 (inositol triphosphate) and ryanodine receptors or acting directly on posttranscriptional mechanisms [20]. Upon signal reception, the pacemaker generates and sustains rhythms that are subsequently diffused to the peripheral organs via output pathways such as the glucocorticoid pathway [21]. Peripheral oscillators exist within the different tissues throughout the organism being present in most cells (Fig. 1) and regulate various biochemical processes [1,22].

Several key characteristics were defined to describe the circadian clock: it is self-sustained or endogenous—the circadian rhythm persists even in the absence of environmental inputs; it is entrainable—the oscillator can be reset or phase shifted by exposure to different time cues (e.g. light) which allows synchronization to the external light/dark cycle; it is temperature compensated—the period of circadian rhythms changes only slightly under different temperatures within the organism's physiological range; and it is able to transmit a time-signal to peripheral oscillators and reset those to the prevailing zeitgeber [23].

The endogenous mechanism that generates sustained oscillations—in peripheral and SCN cells—is constituted by a gene regulatory network. A set of 14 genes forms the core-network of the mammalian circadian clock, the core-clock network (CCN), that accounts for the generation of circadian rhythms within individual cells [19] (Fig. 1, lower panel). These elements are necessary for the robust generation of oscillations, which can occur in the absence of external inputs. This characteristic justifies the designation of core-clock and was first demonstrated computationally [14]. These genes are members of the *Per* (period), *Cry* (cryptochrome), *Bmal* (brain and muscle ARNT-like protein), C*lock* (circadian locomotor output cycles kaput, NPAS2 in neuronal tissue), *Ror* (RAR-related orphan receptor) and *Rev-Erb* (nuclear receptor, reverse strand of ERBA) gene and protein families. All elements in this network interact via positive and negative transcriptional and translational feed-back loops [14].

During the early time of the circadian day, the heterodimer complex CLOCK/BMAL1 is formed and regulates the transcription of all other genes within the CCN. This is achieved via binding of the CLOCK/BMAL1 complex to E-Box sequences within the promoter regions of the target genes, *Ror*, *Rev-Erb*, *Per* and *Cry* [14,19].

The network can be seen as the interconnection of two larger loops, the PER/CRY (PC) loop and the REV-ERB/*Bmal*/ROR (RBR) loop [14]. In the PC feed-back loop, following transcriptional activation of *Per 1*, *2*, *3* and *Cry 1*, *2* genes and translation of the respective proteins, PER and CRY family members form complexes (PER/CRY) which translocate into the nucleus and inhibit CLOCK/BMAL1 mediated—transcription, regulating the expression of all core-clock genes (Fig. 1, lower panel).

PERs and CRYs were first thought to drive alone the sustained oscillations in the clock however, recent studies using fine-tuned experimental and mathematical models identified the second feed-back loop, the RBR loop to be necessary for the robustness of the system, and furthermore, showed that this loop is able to generate oscillations on its own [14].

After the binding of CLOCK/BMAL1 to promoter regions of *Rev-Erb α*, *β* and *Ror α*, *β*, *γ*, these genes are transcribed and subsequently translated. The resulting proteins compete for RORE elements within the *Bmal1* promoter region and hold antagonistic effects, thereby fine-tuning *Bmal1* expression (as depicted in the network in Fig. 1). Both the PC and the RBR loop are able to produce rhythms in gene expression, independently, but need to be interconnected to robustly generate oscillations with a period of circa 24 h [13,24].

Similar auto-regulatory genetic networks were also described for other organisms, including *Drosophila* [25], zebrafish [26] and cyanobacteria [27], although most likely from diverse evolutionary origins [28]. The intricacy of the different clock networks is variable in its complexity, ranging from a three elements network in cyanobacteria to the above described 14 genes network for mammals. It is worth noticing that non-transcriptional clocks were described in red blood cells which may generate oscillations in the absence of transcription [29], but these are beyond the scope of this review.

## 3 Circadian control: the search for new clock genes

The CCN is meanwhile described in detail and the focus of research is now to identify the targets of these circadian genes, the so called clock-controlled genes (CCG). Theoretical and experimental approaches are used to extend the network [10]. Recent work from our group resulted in the generation of a comprehensive network of clock-controlled genes, which we then extended using only bioinformatics approaches, to 118 new clock-regulated genes [9]. These newly identified genes are involved in well-known mechanisms, such as immune defence, apoptosis and metabolism, as well as the regulation of several miRNAs [10]. The deregulation of these genes is found to be associated with cancer development and progression [30].

Peripheral oscillators are present in different tissues and expression of CCGs is thought to be tissue-specific. Although it was first though that 10% of the genome is under circadian control [19], recent studies on mouse tissues indicate that roughly 50% of all genes oscillate in a circadian manner [31], reinforcing the idea of a tissue-specific regulation. Although given the increasing number of tissues and cells under circadian investigation and the improvement towards the techniques used to detect also low expressed RNAs and proteins, the number of reported genes showing an oscillating phenotype may increase.

Given the considerable number of genes which is under circadian control it is of no surprise that many of the most important systems and processes are circadian regulated. Indeed independent studies point to circadian influence on the immune system [32], the metabolism of different metabolites [33], bone formation [34], sleep–awake cycles [35], memory consolidation [36], blood pressure, body temperature, cell division and proliferation [37], hormone regulation [38], apoptosis and senescence [39]. Therefore, as a consequence of a deregulated clock a number of pathologies may arise and are described below.

## 4 Circadian models: from the bench to the computer

The choice and design of an appropriate model to address the question under study is the first step for a successful project, whether theoretical or experimental. A model allows the organization of information and facilitates the understanding of the hypothesis under investigation. A model can start as a simple diagram where defined correlations between genes and proteins are represented and used to illustrate ideas or can be further developed into a complex set of mathematical equations. Moreover, it can be conceptual or contextual, depending on the implementations of a defined context; diagrammatic or computational; qualitative or quantitative; discrete, continuous, stochastic or deterministic [40]. Independent from the choice of model, there are some characteristics that a circadian model must have: be rhythmic and with a circadian period which must be temperature-compensated and entrainable to external rhythms; the amplitude for the rhythms must drive outputs and not damp under constant conditions [40].

Diverse models have been created to simulate the circadian clock in different species (Fig. 2). Mathematical differential-equation models have been developed for mammals (*Mus musculus*) [14,41,42], insects (*D. melanogaster*) [43–46], plants (*Arabidopsis thaliana*) [47–49], *Ostreococcus tauri* [50,51], fungi (*Neurospora crassa*) [52–54], and cyanobacteria [55,56].

So far, focus is set on modelling of the core-clock in an attempt to elucidate its characteristic properties, such as self-sustained oscillations, robustness, synchronization among cells and entrainment to external inputs. Other models rather focus on investigating alterations in clock properties upon perturbations of the model parameters, which allows the simulation of pathological situations where the clock is known to be deregulated [9,57].

More comprehensive models may also be used to simulate the circadian network at a systems level, by including clock-controlled genes (CCGs). A large fraction of genes is subject to tissue-specific circadian rhythms [31,58,59], representing a vast diversity of pathways [10,31]. Such model architecture would thus enable the investigation of circadian effects in a tissue-specific manner.

When creating a model, a decision has to be made regarding the choice of variables and processes. Reaction times play a particularly important role in modelling circadian systems. In addition to direct gene regulations via transcription factors, phosphorylation, complex formation and other types of proteins or mRNA modifications might also be of importance, depending on the scope of the model and the techniques used.

## 5 Experimental models

Experimental models for the circadian clock include *in vivo* and *in vitro* systems.

Diverse organisms from unicellular ones to vertebrates are objects of *in vivo* circadian studies, as represented on the left panel of Fig. 1. Lower complexity organisms are used to understand basic circadian mechanisms which can then be extrapolated to more complex organisms. Cyanobacteria [60] are well-studied organisms as well as *D. melanogaster* [61], *N. crassa* [4] and *A. thaliana* [62]. Within the vertebrates, the mouse is the best studied system [19]. Its similarity to the human circadian structure allows the usage of this system to investigate clock-related pathologies. Additionally, a few circadian studies were reported in zebrafish [63]. Due to its characteristics, namely the transparency of the organism and its relatively large embryos, which make this system highly interesting for life microscopy studies, as well as real time circadian measurements, the usage of zebrafish as a circadian model will certainly become more popular.

Alterations in the circadian phenotype can be studied by analysing the behaviour or activity of an animal, under different conditions, such as different light/dark cycles or mutations in CCN genes. These may influence the circadian output and lead to different periods of activity as happens with Cry1^−/−^ or Cry2^−/−^ mice that develop a shorter or longer period, respectively [64] or even absolute loss of the circadian rhythm as it was observed for Bmal1^−/−^ [65] and Clock^−/−^Npas2^−/−^ [66] mice. Also bioluminescence approaches can be used *in vivo* to report real-time expression of genes or proteins with a role in the circadian clock [67].

*In vitro* experiments commonly use a luciferin reporter system, in primary cells or cell lines, to measure activity of clock genes in real time [61,67,68]. In addition, the levels of proteins or RNA can be quantified at multiple time-points to further validate and mechanistically address circadian observations. A summary of these different methodologies is depicted in Fig. 2.

## 6 Computational models

Mathematical models aid experimental design and serve as a substantial complement to experimental approaches, regarding both the complexity of behaviour and the high density of interconnections in the gene regulatory networks involved [69].

Of major importance for the characterization of gene regulatory network models is the identification of the network structure. Various enhancer or repressor binding sites may be present in the promoter region of a gene. The identification of binding sites using for example ChIP-seq data involves a sequence of classical bioinformatics applications [70]. The reads identified by ChIP-seq yield a fuzzy picture of counts over base pair position, from which peaks are selected in a sophisticated procedure named “peak calling” and used as a source to search for overrepresented sequence motifs [71]. Motifs, such as the E-, D-boxes and RREs, known binding sites for core-clock genes, can be represented as a simple sequence or position weight matrix (PWM). The matrix can subsequently be matched to positions in the genome considering the statistics of random occurrences and associated to promoter regions of specific clock-target genes [72].

The key characteristic of the clock network is its ability to generate rhythmic output. However, the complete picture of causes behind the varying observed rhythm is often difficult to grasp intuitively. Depending on different conditions, such as binding strengths of the elements involved (genes and/or proteins), degradation speed and temporal delays, the behaviour of a certain network may differ, exhibiting different phase-times, displaying or not displaying oscillations, harmonic behaviour or even chaos [73–75]. Therefore, different conditions and a large amount of interconnected processes have to be taken into account in order to explain the varied behaviour of the systems, which makes computational and mathematical methods and analyses complex.

Theoretical analyses revealed that, when assembling a mathematical model, a number of rules apply which enable and further facilitate oscillations. These comprise a temporal delay and a nonlinear response in influencing effects, as well as a negative feedback loop [45]. The latter may be present in different forms constituted by combinations of activating and inhibiting processes [76]. Additionally, positive feedback loops could be shown to contribute to reducing the demands on the strength of nonlinearities, which, if too high, are regarded as inappropriate for biological systems [45]. In biochemical reactions, the strength of nonlinearities commonly corresponds to the size of Hill coefficients [77].

The first biochemical, negative feedback oscillator model reported—the Goodwin oscillator—contains three interconnected components with specific production and degradation rates [78]. Since then, a growing amount of more complex and more close-to-biology models was established as indicated above, which comprises more genes and explain subtle experimental observations.

Within the different mathematical models for the circadian clock, there are particular aspects under study, which lead to specific phenotypes of interest: oscillations, with a period of around 24 h, are continuously observed even after elimination of all zeitgeber signals and have to be robust against diverse changes in a cell's molecule composition [79]. Other models—coupled oscillators—focus on the synchronizing influences on the clock network and the coupling of numerous clocks [80]. It may also be of interest to investigate the properties of circadian systems under the influence of different intertwined synergistic mechanisms, such as potential rhythm generating feedback loops [14]. To examine the requirements and interrelations of such complex systems in detail, mathematical models are used as a tool to connect experimental observation with mathematical assumption, thereby allowing to answer specific questions about unclear and sometimes counterintuitive systemic effects.

## 7 A mathematical description of the circadian system

Traditionally, circadian clock networks are commonly modelled using ordinary differential equations (ODEs) to describe gene expression changes per time [41,42,81]. This approach characterizes time delays implicitly using the other kinetic parameters of the model. However, it is also possible to introduce time delays explicitly using the lesser known delayed differential equations (DDEs) [73,82]. In case of not well characterized time determining mechanisms, as is the case for core-clock proteins, DDEs may serve in overcoming the missing information and simplify the model. The model then becomes infinitely dimensional, however, numerical methods such as DDE-extensions of the well-known Runge–Kutta methods for iterative approximation of ODEs may be used to solve the equations efficiently [83,84].

Differential equation models are dynamical and thereby require diverse kinetic parameters, which often results in time consuming analyses. If the kinetic experimental parameters for gene regulatory models are not known, or if the network becomes too large and computational expenses of detailed simulations are too high, the usage of discrete approaches may be an alternative [85,86]. Such approaches can be discrete in both time and/or concentration levels. An example are Boolean gene regulatory networks, where each gene is allowed to be in either an ON or OFF state. Transitions between the states are then determined by logic functions [87]. Boolean models have been used to simulate basic properties of the core-clock in *A. Thaliana* [88].

In addition to the Boolean and differential equation models mentioned above, there are also discrete models, which allow a fixed number of concentration levels for the different elements (gene/protein). There are many mixtures between discrete and continuous aspects of the modelling and different ways of incorporating time delays in each [89]. Regarding the complex dynamics of circadian clocks, in contrast to other networks such as signal transduction pathways, discrete models are not very common.

Stochastic modelling techniques that make use of the Gillespie method have been applied to model circadian systems and may for example be helpful in studying the effect of noise [90–92].

Although mathematical models have proven to be helpful in understanding biological systems, there are natural limitations due to the different abstractions and model assumptions of these approaches and care has to be taken about which answers may be expected from each type of model.

## 8 Data preparation and model fitting

Starting from experimentally retrieved data to the construction of a mathematical model, a number of steps need to be carried out. It is crucial to extract meaningful information from the data by dealing with experimental noise and inaccuracies using the repertoire of statistical procedures available [93,94]. When dealing with very large data sets, bioinformatic techniques are necessary to compute large amounts of digital data efficiently [95].

Oscillatory changes in gene expression may be obtained experimentally via luciferase or fluorescence assays [61,68]. The retrieved data consists of a series of time-point measurements showing, directly or indirectly, the circadian phenotype of the gene under investigation. To characterize the oscillations precisely, a rhythmic function is usually fitted to the data. This implies a modelling assumption, assuming an abstract pattern behind the observed phenomenon, self-sustained oscillations and/or a fixed period.

Simple combinations of trigonometric functions are commonly assumed: A function like,

$$y=a*\sin\left(\frac{2\pi }{24}*x\right)+b*\cos\left(\frac{2\pi }{24}*x\right)+c\text{,}$$

can be fitted by linear regression [96]. Such a function can then for example be used to determine phase times by extracting its peaks. To allow for a higher variety of curve shapes, the above function can be extended by harmonic terms with half of the period or in case of damped oscillations a damping factor can be added. The COSOPT algorithm [97] implements such an approach acting in the time-domain. A variety of alternative functions may be chosen for appropriate fitting. For variable periods or amplitude data, wavelets may be a choice to handle this volatility [98]. In addition to approaches acting at the time-domain level, other methods exist that act in the frequency domain, [99,100]. An algorithm making use of the advantages of both types of approaches was suggested as well [101].

CircaDB is a database [102] that provides many gene expression time courses for circadian genes. The maSigPro package for Bioconductor [103] may be used to identify differentially expressed genes in measured time-courses. Identification of the period of circadian time courses is for example possible with the BioDare online service [104].

A network structure and additional time-series data may serve as the basis of a circadian model. The network structure is used to establish a set of equations and the time series for fitting in order to determine the parameter values of the model. Particularly kinetic parameters, that describe the production and inhibition rates of RNAs and proteins, are commonly obtained from the fitting procedure. Other parameters, such as degradation rates of RNAs and proteins can more often be found in the literature. Even though a parameter can be chosen manually to reflect experimental measurements, fitting in a small and appropriate range may be beneficial for fine tuning. As data usually possesses a certain range of accuracy, the respective parameters can be allowed to vary in a comparable range of values, mirroring these uncertainties. Incorporation of as much experimental information as possible reduces the risk of arbitrariness. Comparison of different repeated fitting solutions may as well help to debunk this pitfall.

For very large and complex mathematical models it might also be necessary to estimate missing parameters using a global optimization algorithm. Various different techniques exist in this field, suitable for different kinds of problems. Fitting of gene regulatory networks is often conducted, using Evolutionary Algorithms [105] or Markov Chain Monte Carlo (MCMC) sampling based approaches, such as Simulated Annealing [106]. These approaches have the advantage that the objective function—a measure of accordance with the data points—does not need to be differentiated and positions in the parameter space can be simply sampled. Evolutionary Algorithms are effective, if the parameters of the model can be sensibly encoded into the “genome” of a solution, such that a “cross-over” step between different individual solutions may be reasonably established. The well-known Gradient Descent Method is suitable to find local minima only, however, strategies may be established that combine population and/or sampling based methods with the use of gradient descent. There is no definite answer which optimization strategy to follow, but a process of trial, error and adjustments is inevitable in this key tuning step.

Following the construction of the mathematical model, additional insights on the biological system may be obtained from the analysis of the same model. The changes of period and amplitude values with variations of the parameter values can be analysed to obtain a picture of the role and importance of the parameters. Such variations can be depicted in bifurcation- and period-diagrams. By displaying both minima and maxima over a range of varying parameter values, bifurcation-diagrams give information about many characteristic features: the value at which the oscillations start, emerging with a transient of damped oscillations, and the increase of amplitude. Also tori or chaotic behaviour may be observed in bifurcation-diagrams. Analysis of the scope of solutions obtained from different fits may help to reveal information about the distribution of physiological parametrizations [107].

Furthermore, such an analysis may be of use to identify the key parameters associated to a certain circadian phenotype, mimicking an observed pathological situation [9,57], and thereby contribute to the identification of possible nodes in the network (genes/proteins) for which perturbations disrupt the clock and help to understand its role in disease.

Of further interest is the linkage of the core-clock to other pathways and so called clock-controlled genes (CCGs). To unravel the embedding of the core-clock into a larger network of clock-controlled genes systems biology methods are used. These include the analysis and integration of different types of large scale data [10,11,108]. Such data comprise gene co-expression, protein-protein interactions, ChIP-seq data, GO terms and KEGG pathways analysis, as well as text mining data [109,110].

## 9 The mammalian circadian clock in disease: when time fails

Comprehensive studies have been carried out to investigate the significance of clock gene perturbations, at the cellular and organismal levels. An extensive RNAi screen in human cells delivered new insights into the effects of clock gene knockdown on circadian rhythms, showing that complex changes within the circadian clock network occur [12,111].

The question as to what are the causes for a disrupted clock is not yet completely answered, although promising candidates have been identified. These vary from external factors like night shift work, variations of day length, melatonin release, exposure to artificial light and to low frequency electromagnetic waves, dietary factors, to internal deregulations at the cellular level, regarding genetic disorders in clock or clock-regulating genes [112].

As the circadian clock is involved in the regulation of numerous biological processes, it is of no surprise, that its deregulations are associated with many different types of diseases. These include sleeping disorders (familial advanced sleep-phase syndrome (FASPS), sleep problems in the elderly) [14,35], neuropsychiatric disturbances (seasonal affective disorder (SAD), bipolar disorders) [1,14], metabolic diseases (diabetes and obesity) [113,114], cardiovascular disorders [112] and cancer development [115]. Additionally, weak response to anticancer treatments was reported in cancer patients with disturbed circadian rhythms, as compared to the ones with normal circadian phenotype [116].

Nevertheless, the mechanisms via which the circadian clock leads to disease remain mostly unclear. One example is the seasonal affective disorder (SAD) where the clock fails to synchronize during winter season due to low light intensities. Consequently, progressing phase delays lead to discomfort up to severe depression [1]. Other pathologies, like the familial advanced sleep phase syndrome (FASPS), are well understood and specific mutations on the phosphorylation sites of the *Per* gene were identified to be responsible for the phenotype [35].

Shift working over long time periods and associated deregulations of the circadian clock seem to be a high risk factor for various diseases including cardiovascular diseases, metabolic syndrome, diabetes and cancer.

The challenge at this point is to discriminate between direct effects of circadian clock disruption and indirect effects due to life style as a consequence of shift work itself [1], since for example a connection between night shift work and metabolic syndrome was also shown [117,118].

The link between an impaired clock and cancer risk was reinforced with several reports [115,119] and, in 2007, the IARC (World Health Organization's International Agency for Research on Cancer) listed “shift work that involves circadian disruption” as a probable carcinogen [116]. Similarly, numerous epidemiological studies linked night shift working to an increased susceptibility to different cancer types. These include colorectal cancer [120], breast cancer [121–125], prostate cancer [126,127], endometrial cancer [128] and non-Hodgkin`s lymphoma [129]. Also impaired melatonin levels, resulting from the disturbed synchronization of the clock with the environment during night shift work, are seen as one of the reasons for the increased cancer risk [130].

Also, mutations in clock genes or changes in their expression levels and rhythmicity were observed in various cancer types in animal models, human cell models, patient samples and population based studies. Additionally, the circadian clock regulates cell cycle, DNA damage responses, ageing and metabolism through different mechanisms. Hence, altered circadian rhythms may also lead to impaired regulation of these processes, leading to tumourigenesis [115].

Further evidence for the role of the clock in cancer was provided by population studies on premenopausal women with variant *Per3* who have an increased risk for breast cancer [131]. Moreover, the expression of all *Per* genes was shown to be disturbed in 95% of examined women with this type of tumour [132].

Concomitant with the results from population studies, *in vitro* studies revealed that *Per1* and *Per2* overexpression leads to growth inhibition and apoptosis, pointing to a tumour suppressor function of the *Per* family [133,134]. In addition to that, overexpression of *Per1* leads to increased DNA damage-induced apoptosis in colon cancer cell lines [133] and growth inhibition and apoptosis in prostate cancer cells [135]. In different human lymphoma cell lines as well as in tumour cells from acute myeloid leukaemia, mRNA levels of *Per2* are downregulated [136]. These results underline the role of PER in tumour suppression. Interestingly, a screening of breast cancer cell lines revealed that more than 50% of the cell lines were hypermethylated on the promoters of *Per1*, *Per2*, *Cry1* or *Bmal1* [137], which hints to an epigenetic mechanism of clock gene disruption [112]. Moreover, disruptions of the core-clock in human cancers are diverse and include deregulation at the transcriptional and post-transcriptional levels and structural variations of clock proteins due to circadian gene polymorphisms [116].

Other components of the core-clock were also reported to be disrupted in different cancer types. Epigenetic inactivation of *Bmal1* can often be found in hematologic malignancies, such as diffuse large B-cell lymphoma, acute lymphocytic leukemia (ALL), non-Hodgkin's lymphoma (NHL) and acute myeloid leukemia (AML). Polymorphisms in *Clock*, *Npas2* and *Cry* genes are often linked to increased risk or recurrence of colorectal and breast cancers, NHL, AML and endometrial ovarian cancer [116]. Mutations in *Npas2* seem to correlate with an increased risk of breast cancer and non-Hodgkin's lymphoma [115,138]. Furthermore, a link between the variant *Cry2* allele and increased prostate cancer risk was shown in a population-based study [139].

The possibility that not only one clock component is involved in tumourigenesis, but rather deregulations of some or all core-clock genes are likely to be present in different cancer types has to be taken into account and was hypothesized by various studies [116].

Taken together, these findings point to a role of the circadian clock in tumour suppression and the deregulation of the circadian system seems to play a role in cancer development and progression.

Still, the results gained from human cell lines, patient samples and animal models are difficult to interpret, as the effects of clock gene disruption and deregulation of circadian rhythms can depend on the experimental model used. Furthermore, it partly remains unclear whether the impact of the circadian clock disruption in cancer development is due to the disruption of circadian rhythms itself or due to other regulatory features of core-clock genes such as regulation of the cell cycle, DNA damage responses and cellular metabolism [115]. Systems biology approaches, bioinformatics methods and computer models can help to contextualize the effects of the molecular clock on cancer development and progression.

The increasing awareness of the importance of the circadian clock in cancer development and progression also led to the development of new treatment strategies which take into account the patient circadian clock and as such the time for treatment (both radiotherapy and chemotherapy times)—chronotherapy. With the help of time dependent drug administration, efficacy and tolerability should be increased, whereas toxicity and side effects should be decreased to a minimum. This effect may be mostly due to the circadian control of metabolic processes, which strongly influence the pharmacokinetics and pharmacodynamics of anticancer drugs.

Indeed, a meta-analysis revealed that a specific chronomodulated chemotherapy protocol led to better treatment response and prolonged overall survival in men with colorectal cancer by 3.3 months [140]. This result was however, not applicable to women underlying the need for gender and patient specific dosage timing protocols. A study to evaluate the advances of cisplatin-based chronotherapy in patients with advanced non-small cell lung cancer did not yield significant differences between the groups regarding the total response rate [141]. Another study on the relapse risk for children with acute lymphoblastic leukemia, shows a dependency of time in terms of event-free survival outcome and favours evening treatment schedules [142].

Similar to chemotherapy, also radiation can be administered according to a time-dependent schedule [143–145]. As an example, morning radiation caused more hair loss than evening radiation in mouse experiments [146]. Likewise in humans, the secondary effects of radiotherapy seem to be day-time dependent, although only a few rather contradictory studies were reported [147,148]. As seen for the chronomodulated chemotherapy approach, also radiotherapy shows individual effects depending on sex, age and other individual patient-factors.

In cancer patients, different circadian biomarkers can be determined, which provide hints into the robustness of the circadian timing system. These include melatonin and cortisol levels, body temperature and rest-activity rhythms, which are often disrupted in cancer patients and can lead to cancer associated fatigue, sleep problems and decreased overall survival [149]. To account for this, therapeutic approaches exist that aim to balance the disrupted circadian timing system in those patients. These include regulation of the sleep-wake cycle, physical activity, light therapy, timed meals and synchronization through chronobiotic drugs [149].

Studies to date are comparably rare and fail to give clear messages with respect to efficient chronotherapy. Reasons for this might be the heterogeneity of different tumour entities as well as the individual circadian profile of every single patient.

## 10 Summary and outlook

During the last decades, much effort has been made to explore the circadian timing system in all its facets. The core-clock has been studied in detail and new clock-regulated genes and pathways have been identified. The consequences of clock disruption have been analysed and the role of the circadian clock in diseases, such as cancer has been explored. Additionally, to date computational models exist that facilitate the prediction of consequences of perturbations in the circadian system.

Yet, the mechanisms driving chronobiology remain unclear. In particular, the distinct roles of clock gene perturbations and circadian rhythm disruption in disease contexts need innovative and interdisciplinary approaches to elucidate the role of the circadian clock in pathological conditions.

The awareness of the fact that for example in cancer, disrupted circadian rhythms lead to poor prognosis and worse treatment responses justifies the need for further research in the field, although much effort has already been made in that direction. There are already a few drugs that target clock genes aiming to restore the disrupted circadian timing system [150]. Studies in the field of chronotherapy highlight the importance of correct time of treatment. Nevertheless, additional research is needed in the field of chronotherapy and the individually timed drug administration should be subject to further studies as the to-date available data point to improved drug response and tolerability. These are certainly exciting times for medical chronobiology, which though at its infancy starts to succeed in demonstrating its enormous potential towards understanding the role of time in human health and disease.
